# Wound area measurement with 3D transformation and smartphone images

**DOI:** 10.1186/s12859-019-3308-1

**Published:** 2019-12-18

**Authors:** Chunhui Liu, Xingyu Fan, Zhizhi Guo, Zhongjun Mo, Eric I-Chao Chang, Yan Xu

**Affiliations:** 1 State Key Laboratory of Software Development Environment and Key Laboratory of Biomechanics and Mechanobiology of Ministry of Education and Research Institute of Beihang University in Shenzhen, Beijing Advanced Innovation Center for Biomedical Engineering, Beihang University, Xueyuan Road No.37, Beijing, 100191 China; 2 China mobile research institute, Xuanwumen West Street No.32, Beijing, 100053 China; 3 Bioengineering College of Chongqing University, Shazheng Street No. 174, Chongqing, 400044 China; 4 Microsoft Research, Danling Street No. 5, Beijing, 100080 China; 5 Beijing Key Laboratory of Rehabilitation Technical Aids for Old-Age Disability, Key Laboratory of Rehabilitation Technical Aids Technology and System of the Ministry of Civil Affairs, National Research Centre for Rehabilitation Technical Aids, No.1 Ronghuazhong Road, Beijing Economic and Technological Development Zone, Beijing, 100176 China

**Keywords:** Wound measurement, 3D, Structure from motion, Least squares conformal mapping, Smartphone

## Abstract

**Background:**

Quantitative areas is of great measurement of wound significance in clinical trials, wound pathological analysis, and daily patient care. 2D methods cannot solve the problems caused by human body curvatures and different camera shooting angles. Our objective is to simply collect wound areas, accurately measure wound areas and overcome the shortcomings of 2D methods.

**Results:**

We propose a method with 3D transformation to measure wound area on a human body surface, which combines structure from motion (SFM), least squares conformal mapping (LSCM), and image segmentation. The method captures 2D images of wound, which is surrounded by adhesive tape scale next to it, by smartphone and implements 3D reconstruction from the images based on SFM. Then it uses LSCM to unwrap the UV map of the 3D model. In the end, it utilizes image segmentation by interactive method for wound extraction and measurement. Our system yields state-of-the-art results on a dataset of 118 wounds on 54 patients, and performs with an accuracy of 0.97. The Pearson correlation, standardized regression coefficient and adjusted R square of our method are 0.999, 0.895 and 0.998 respectively.

**Conclusions:**

A smartphone is used to capture wound images, which lowers costs, lessens dependence on hardware, and avoids the risk of infection. The quantitative calculation of the 3D wound area is realized, solving the challenges that 2D methods cannot and achieving a good accuracy.

## Background

The measurement of wounds is an important component in the field of clinical research, the accuracy of which influences doctors’ diagnosis, treatment and research programs directly [1, 2]. In the clinical field, the wound area is considered as an effective and reliable index of later complete wound closure [3]. It also plays a role in drug evaluation and research of wound healing characteristics [4]. Moreover, it can help doctors with wound classification, treatment strategy selection, and propelling the treatment technology forward [3]. Cardinal M et al. [3] show it is a strong predictor of venous leg ulcers healing by tracking the area of a skin wound within 12 weeks. Lavery LA et al. [1] show that the diabetic foot wound area between the first and fourth week can be used to predict the healing effect after 16 weeks, and to assist with the evaluation of treatment and drug use.

The wound measurement method has undergone a transition from 1D to 2D, and then 2D to 3D. The traditional 1D ruler method [5] for measuring wound areas is simple and widely used. It measures the external rectangular of wound width by ruler, flexible rule, or adhesive ruler, and then multiples the wound’s external rectangular width to obtain the wound area. Rahul S et al. [6] show that the measurement result of the ruler method is nearly 150% of the actual area, which is very inaccurate, and it is tedious and time-consuming. The 2D method based on image segmentation [7] is a mature method. It uses a 2D image segmentation and adhesive scale to measure wound areas. Yang [8] have developed a wound surface area calculation method using digital photography, and they investigate its error rate. However, this kind of method has drawbacks such as: (1) Given the existence of human body curvatures, a 2D method is difficult to express in the whole shape of a wound, so as to get the correct area value. (2) The 2D method can be greatly affected by camera angle, and the use of different angles may generate different results. Recently, Foltynski [9] have proposed the Planimator app, which was a correction method of area measurement based on calculated camera tilt angle and the calculation of calibration coefficient of linear dimensions as the weighted average. It overcomes the large error caused by the shooting angle in the 2D measurement, but it still cannot overcome the 2D measurement problem caused by the large body curvature. Meanwhile, when disposable paper rulers are used for area measurement with the Planimator app, some deviations from the true area value may occur when the ticks at these rulers are placed at the wrong distances. On the theoretical level, Zhang B [10] proposes a stereo vision 3D method to measure wound areas, but he does not implement it. Sirazitdinova et al. [11] present a conceptual design of a system using inexpensive consumer level hardware for 3D wound reconstruction. Images are recorded using the interactive app running on the mobile device. The data is transferred to the operational server and processed on it. The resulting data can be shown to the patient and to the clinician. They provide a convenient wound measurement solution that allows patients to receive professional guidance on their injuries at home. However, at present, this is only a conceptual stage and has not been implemented. Further experiments are needed to prove the effectiveness of this scheme. Chen et al. [12] present an efficient and effective 3D surface reconstruction framework for an intra-operative monocular laparoscopic scene based on SLAM. The 3D geometric information of the surgical scene allows accurate placement AR augmentations based on 3D calibration. However, their method is a 3D reconstruction of endoscopic surgery, which does not meet our application scenarios. SLAM is more suitable for objects with rich geometric texture. It is easy to lose frames when rotating, and the point cloud in the map is also very sparse. Therefore, it is not practical for scenes that need to accurately measure the wound area. Huang [13] present a new solution to surface area measurement of vitiligo lesions by incorporating a depth camera and image processing algorithms. They use Kinect V1 or Kinect V2 to capture data. Then the segmented lesion area is calculated using depth data through a software component. Their solution shows good performance in the smooth part of the human body. However, if a huge block of the depth image is missing depth information, the accuracy of area measurement will be compromised.

In recent years, the resolution of smartphone cameras has been getting higher and higher, and now it can reach tens of millions of resolutions, which is enough for most photo-taking scenes. Early smartphone image technology focuses on how to present sharper picture quality. With the development of camera hardware and the universality of smartphones in people’s daily lives, the development of smartphone image technology is shifting to focus on how to use images more effectively. Masiero A et al. [14] have developed a mobile mapping system (MMS) using smartphones, enabling low-cost devices to build reliable MMS. Gatys LA et al. [15] introduce an artistic neural algorithm, combining images taken by smartphone with many famous art works. Liu S et al. [16] propose a method to automatically track facial markers using smartphones. This work inspires us to use the images acquired by smartphone to establish a 3D model of body surface wounds.

The structure from motion method (SFM) has been actively researched by scholars. By analyzing the motion of the object, it can obtain 3D information from 2D images. Since its request of images is very low, SFM can use images taken at random sequences for 3D reconstruction. At the same time, it can save on camera calibration steps in advance, and it has strong robustness. This inspires us to use SFM to implement 3D reconstruction of the body surface, and then to calculate the wound area.

**Fig. 1:**
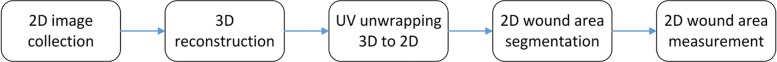
The flow of our method

In this paper, we propose a 3D wound area measurement with smartphone images. The method goes through the process of 2D to 3D to 2D. The definition of 2D to 3D to 2D is as follows: first, we collect 2D images of tested bodies by smartphone, and construct a 3D model using these 2D images; second, we unwrap the UV (Texture coordinates usually have U and V coordinate axes, so called UV coordinates.) map of the 3D model to make it into the 2D plane; finally, we use interactive image segmentation and scale conversion to extract and measure wound areas. The flow of our method is shown in Fig. 1.

Our method provides a complete set of methods for measuring wound area. Since the 3D reconstruction method based on 2D images is adopted, it avoids the situation of frame loss in SLAM real-time reconstruction, making the whole method more practical. At the same time, we convert the 3D model to the 2D plane by LSCM algorithm, and then measure the wound area through the conversion between pixels and real length, which solves the challenge of directly segmenting the wound on the reconstructed 3D model. Moreover, we have verified the accuracy, practicability and effectiveness of this method through clinical experiments.

The contribution of our work is as follows:

1. The smartphone makes it very convenient and quick to capture images of wounded body parts. Our method avoids wound infection, and its sampling is simple and has limited device dependence.
2. We process a novelty pipeline of 2D to 3D to 2D procedure. It overcomes the difficulties of shooting angles, human body curvature, and disabled 3D segmentation.
3. We demonstrate the efficiency and effectiveness of our method by calculating wound areas.

## Related work

Since 3D reconstruction and 3D unwrapping are very important processes in our work, the related work can be divided into three broad categories: (1) wound measurement equipment, (2) 3D reconstruction methods and (3) 3D unwrapping methods.

### Wound measurement equipment

The Visitrak [17] is an electronic device that manually tracks wound boundaries for wound measurements. Users first use the film coverage method to draw out wound borders and then put the film in a Visitrak transparent plate. A pen is used to draw borders in the device interface, and the area value of the wound is automatically calculated with the equipment using the Kundin formula [18]. It can cause pain and risk wound infection, even as it reaches 93% accuracy [19].

The MAVIS [20] uses the color coding principle to realize 3D measurement. It uses a CCD camera to record a set of alternate colors, which is projected onto the wound at about 45 degrees. Then according to the calibrated camera focus, a known location projector and the light intersection of the beam, the geometry of the wound surface is rebuilt to calculate the area. However, the MAVIS is large and expensive, which is difficult to widely use in clinical scenarios. At the same time, in the wound area <10*cm*^2^, the MAVIS error is above 10%.

The Silhouette mobile [21] includes a hand-held computer and an integrated high-resolution digital camera with an embedded laser. The laser launches two beams of light on the edge of the wound, then the Silhouette mobile generates the wound in a 3D model based on the surface topography. The Silhouette mobile can reach 95% accuracy for diabetic foot wounds. However, this expensive Silhouette mobile cannot be applied to telemedicine, and it needs to collect data through a visible laser.

### 3D reconstruction methods

The stereoscopic light method takes multiple photos at the same angle and under different lighting conditions to reconstruct a 3D model. The simplest stereoscopic light method uses three light sources to illuminate the object in three different directions, opening only one light source at a time. It uses three comprehensive photos and the perfect diffuse to work out the gradient on the surface of the object. Then the 3D model is obtained after integrating the vector field. Basri R et al. [22] realize 3D reconstruction under the unknown condition of light source. Hernandez C et al. [23] further propose the use of colored light for reconstruction. However, the stereoscopic light method needs to know the exact location and direction of the light source, so it is difficult to apply in real life.

The stereo vision method [24] is another commonly-used 3D reconstruction method. In concept, this method simulates human eyes to perceive images. It mainly includes three ways of obtaining distance information: directly using the rangefinder, predicting 3D information through a single image, and restoring 3D information by using two or more images on different viewpoints. By simulating the human visual system, it obtains the position deviation between the corresponding points of the image based on the parallax principle, and recovers 3D information.

SFM is used to detect matching feature points in an image in order to restore the position relation between the cameras. Harris C et al. [25] propose the definition of the corner point, and Shi J et al. [26] improve on this and propose a better angle extraction method. The state-of-the-art method of extracting and matching feature points is the scale-invariant feature transform method (SIFT) [27]. Besides the SIFT method, researchers have also proposed some faster methods, such as principal component analysis scale-invariant feature transform (PCA-SIFT) [28], gradient location-orientation histogram (GLOH) [29], and speed up robust features (SURF) [30]. These proposed algorithms are faster than the SIFT method in terms of speed, but weaker in terms of both stability and accuracy. Therefore, the SIFT method is still the best option when there is not much requirement for computing speed. The image demand for SFM is very low, so it can reconstruct a 3D model using video or even randomly shot image sequences. At the same time, the image sequence can be used for camera self-calibration eliminating predetermined steps.

### 3D unwrapping methods

A heuristic method for triangulation flattening is proposed by McCartney J et al. [31]. It uses a triangle list to describe the 3D surface flattening algorithm for 3D unwrapping. The method is based on an optimal local positioning of projected nodes and a sequential addition of the nodes. It incorporates an energy model in terms of the strain energy required to deform the edges of the triangular mesh. It is efficient and produces good results for nearly planar surfaces. However, the method does not guarantee the preservation of the metric structure of the 2D mesh or even its validity.

Eck et al. [32] suggest the use of harmonic maps to generate the 2D projection of the 3D model. It is based on the approximation of an arbitrary initial mesh by a mesh that has subdivision connectivity and is guaranteed to be within a specified tolerance. The method produces approximations of good quality, and provides an accurate mapping function. A major disadvantage of the method is that it requires the boundary of the 2D mesh to be predefined and convex. Another drawback is that the method does not guarantee the validity of the resulting flat mesh, and the method requires the boundary of the 2D mesh domain to be predefined and convex.

The least squares conformal mapping method (LSCM) [33] is a method from polygon mesh to texture mapping, which can map the shape of a 3D model to a 2D texture and is relatively undistorted. The method is robust, and can parameterize large charts with complex borders. It introduces segmentation methods to decompose the model into charts with natural shapes, and a new packing algorithm to gather them in the texture space. By using the map as a guide when creating a new 2D image, the colors of the 2D image can be applied to the original 3D model.

## Results

### Comparison with the stereo vision method

**Fig. 2:**
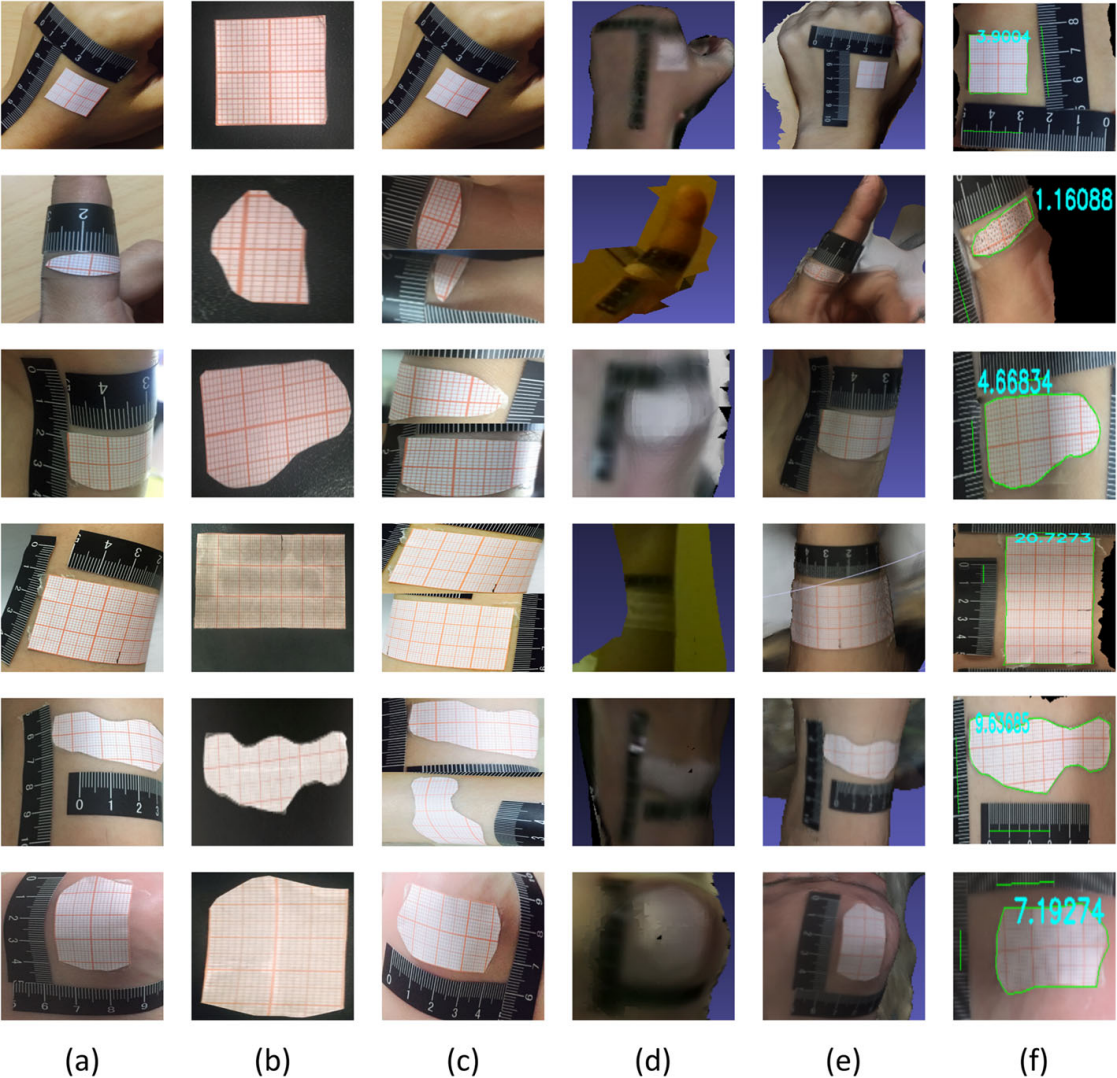
3D reconstruction comparatione of simulated wounds. **a** Images captured by smartphone. **b** Ground truths. **c** Looks of 2D method. **d** 3D model by stereo vision. **e** 3D model by ours. **f** Calculated results of our method. The calculated results of stereo vision is unavailable, so we have to make the results empty here

An example of 3D reconstruction results is shown in Fig. 2. For the wound part based on stereo vision reconstruction, only the fuzzy shape of the wound can be seen. Even the shape of the part cannot be seen clearly, and the wound area cannot be calculated through it. However, for the wound part based on SFM reconstruction, the wound shape can be clearly seen, and its area can be calculated through our method.

SFM obtains the depth information of an object by building the relationship among natural image sequence. It then reconstructs a 3D model of the wound. Compared to other common methods like the stereoscopic light method and the stereo vision method, this method does not require pre-calibration [24] or a special environment [20]. It is a good method of reconstruction in the field of computer vision.

The feature match results play a vital role in building the relationship of natural image sequence. We use SIFT characteristics [27] to match features. Compared to the traditional Harris [34] and KLT characteristics [35], it has immutability towards rotation, scale-zooming, and brightness variation, as well as stability towards visual angle, affine, and noise variation.

### Comparison with 2D measurement

**Fig. 3:**
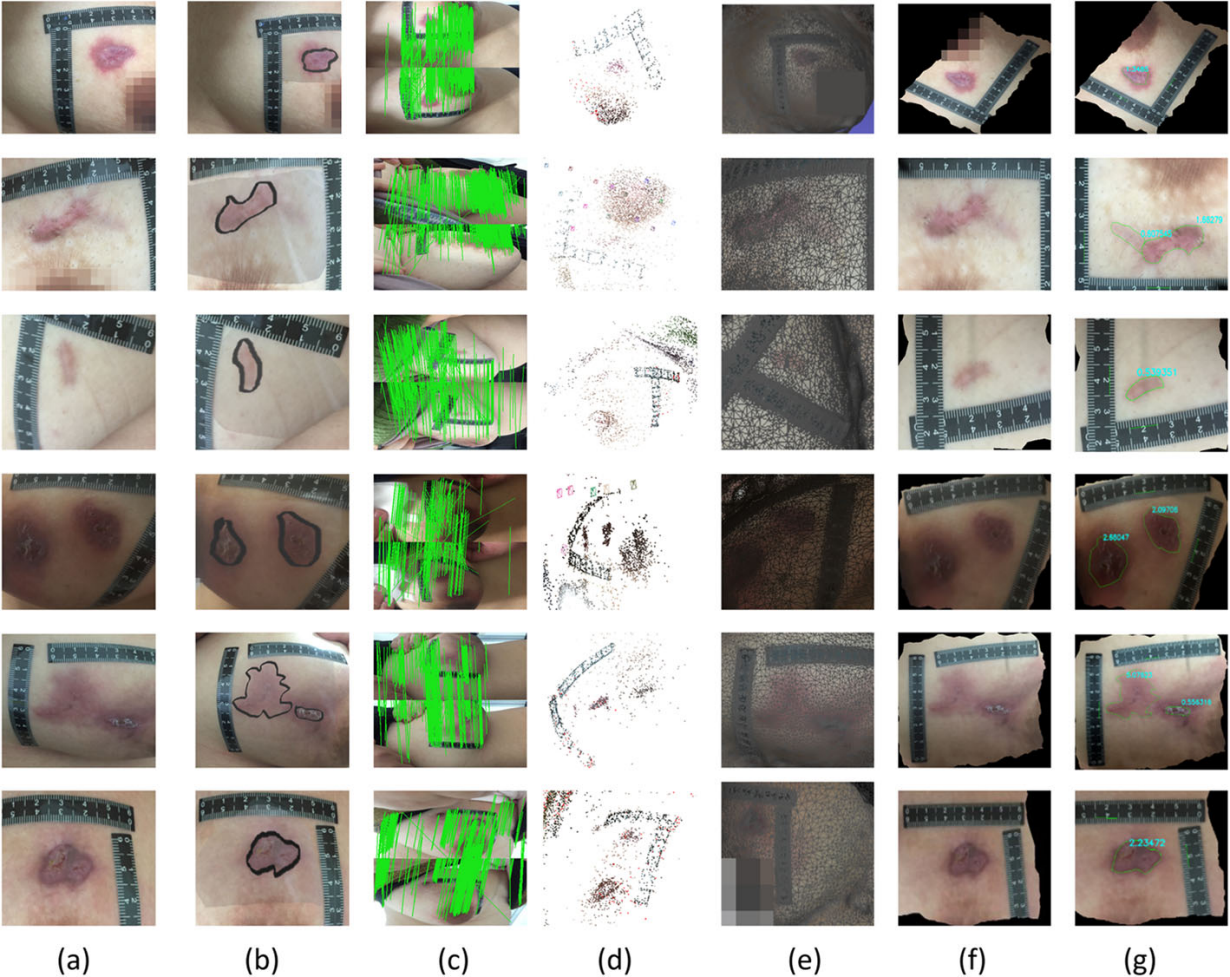
Clinical experience result. **a** Images captured by smart-phone. **b** Ground truths. **c** Results of feature matching. **d** 3D reconstruction results by SFM. **e** Results of networking. **f** Results of unwrapped images (2D). **g** Calculated results of our method

**Fig. 4:**
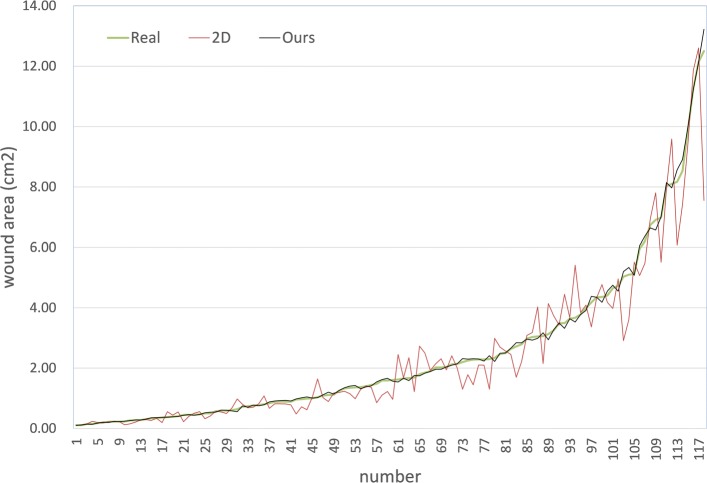
The line chart of ground truth, 2D measurement and our measurement

**Fig. 5:**
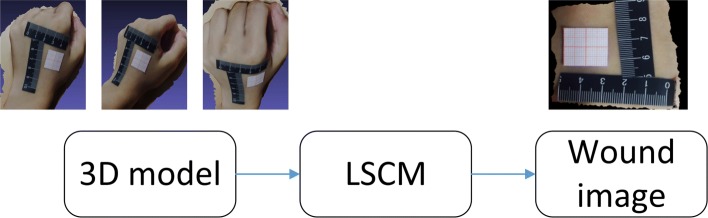
Regression analysis plot of 2D method

**Fig. 6:**
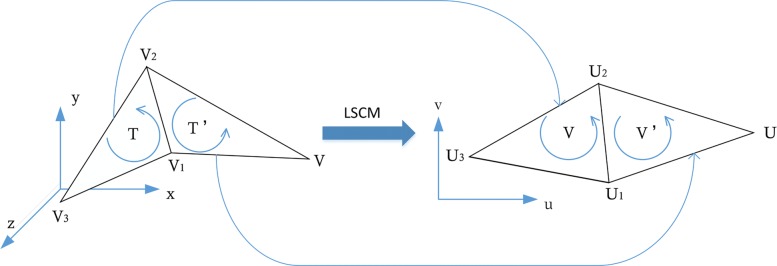
Regression analysis plot of our method

**Fig. 7:**
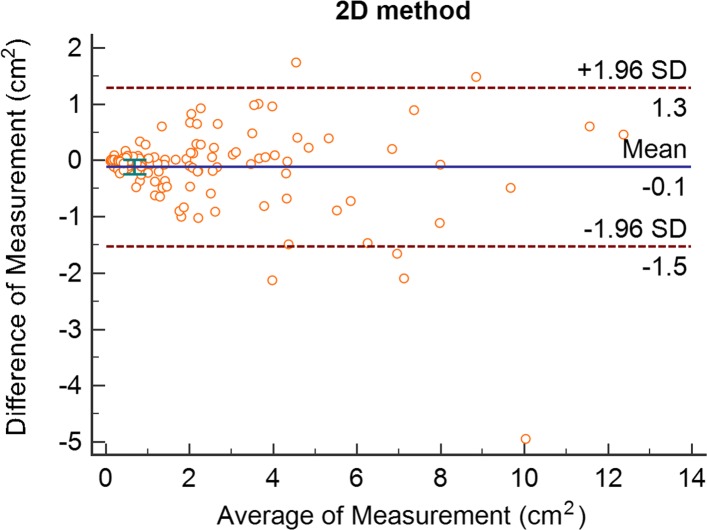
Bland-Altman plot of 2D method

**Fig. 8:**
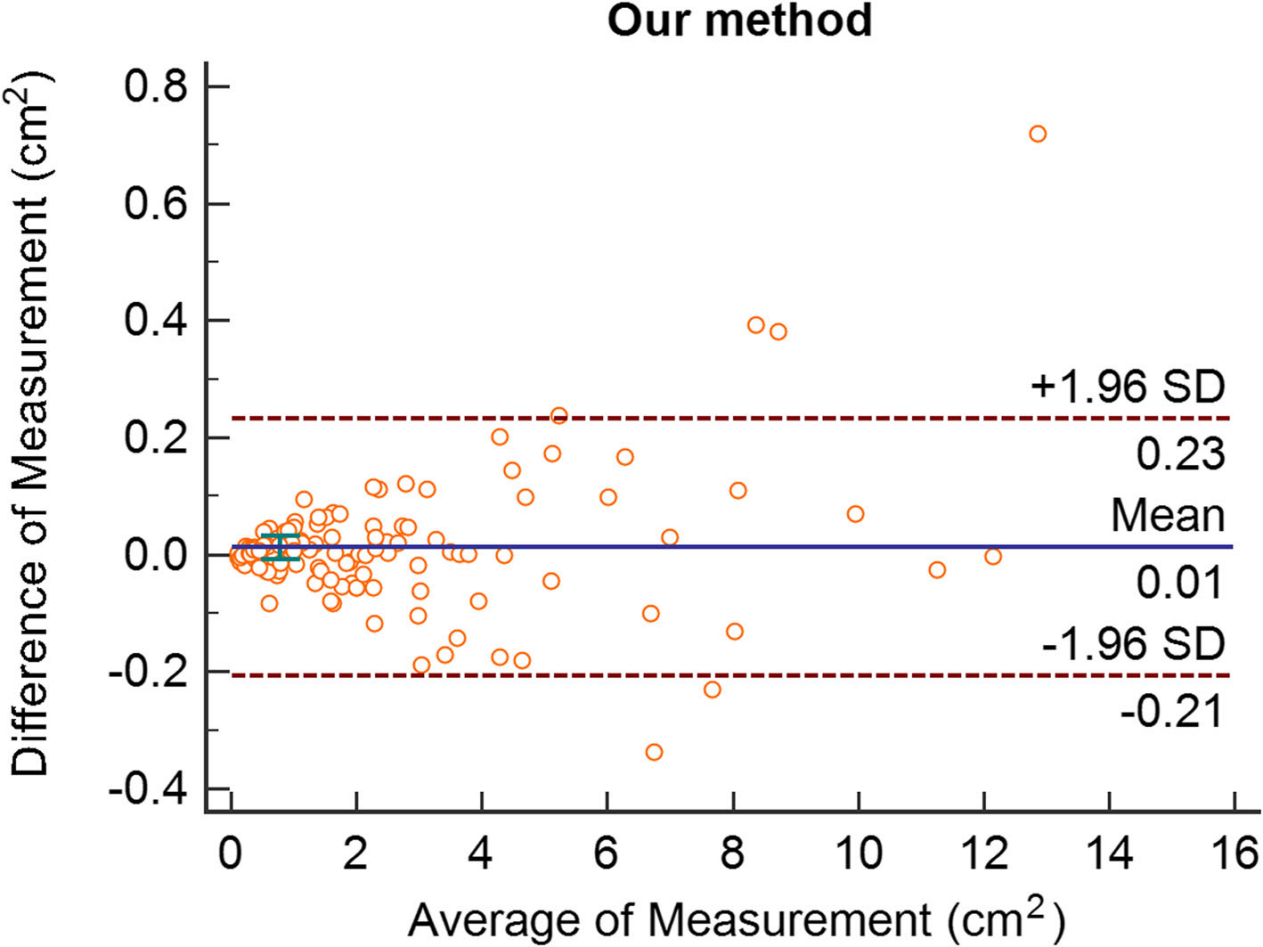
Bland-Altman plot of our method

**Fig. 9:**
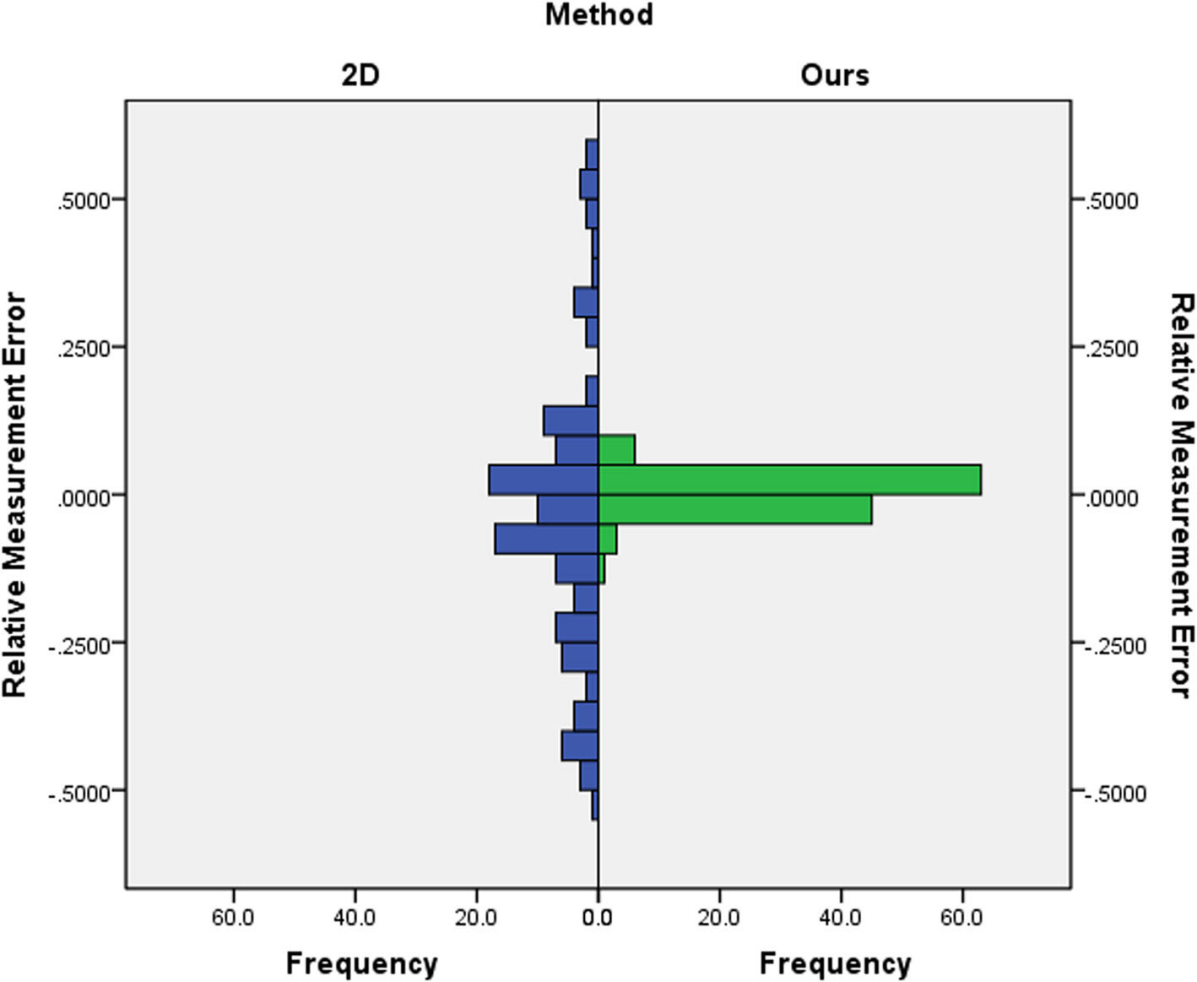
Distributions of relative measurement error

**Fig. 10:**
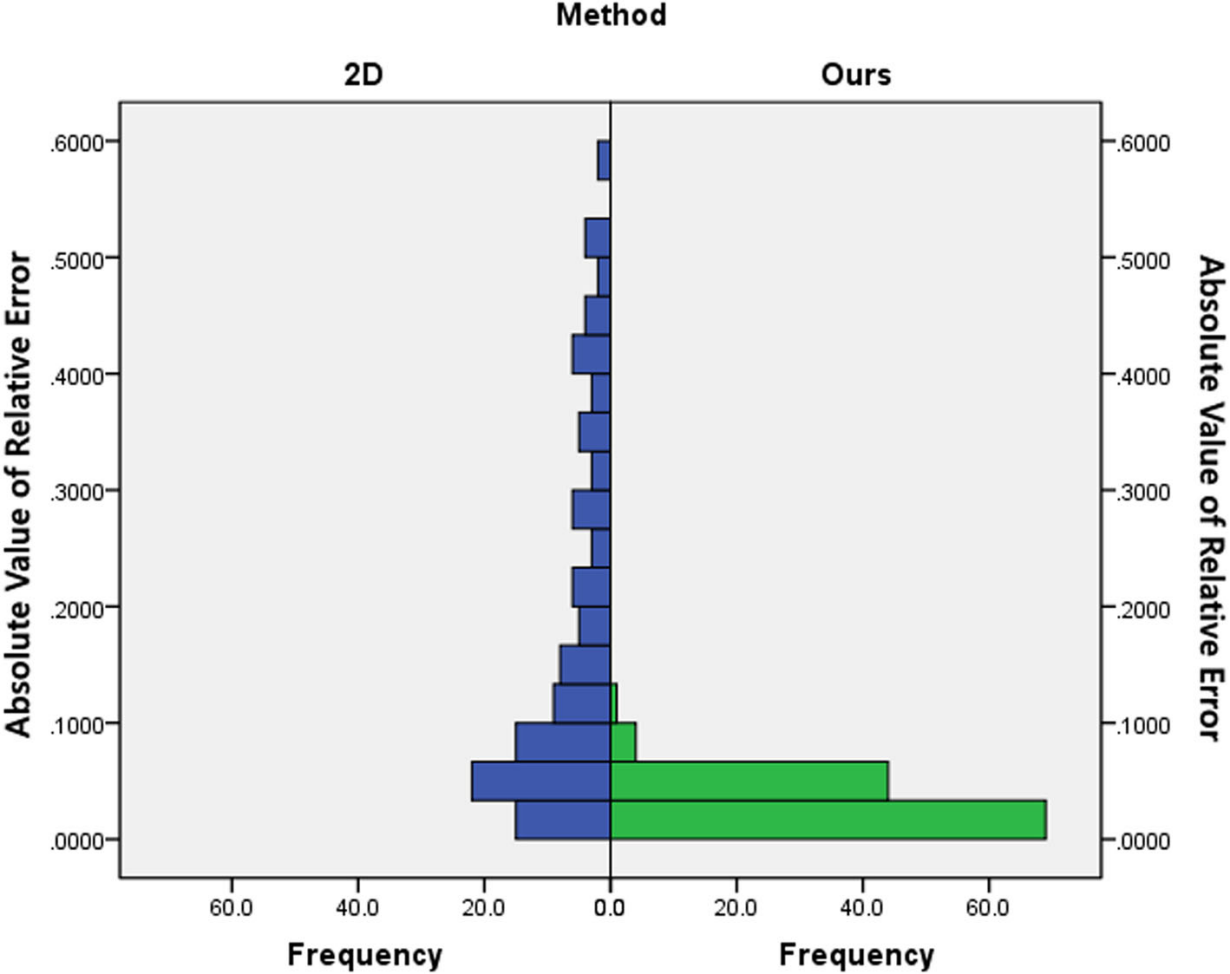
Distributions of absolute value of relative error

**Fig. 11:**
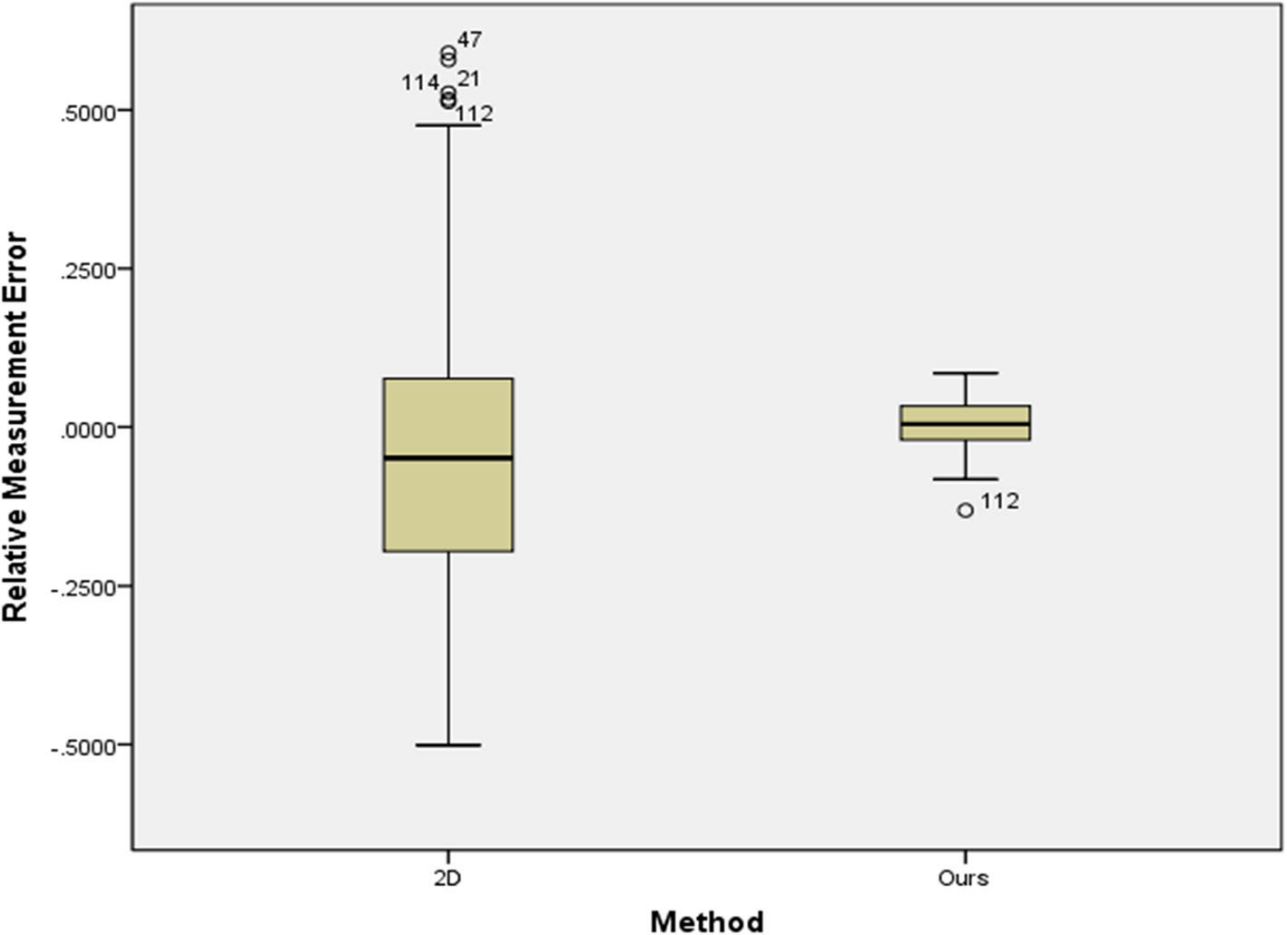
Box-plot of relative measurement error

The experiment results of our method are compared with the 2D measurement result to evaluate the accuracy of our method. The example of area calculation results in our methods are as shown in Fig. 3. The results for the wound area are calculated using our method and the 2D method, with real values shown in Table 1. And the statistical index of Pearson correlation, standardized regression coefficient and adjusted R square are listed in Table 2. The 2D measurement values and the measured values of our method are compared in the line chart, as shown in Fig. 4. The regression curve of 2D method and ours are shown in Figs. 5 and 6 respectively. And the Bland-Altman plot of the 2D method and ours are shown in Figs. 7 and 8. The distribution of relative measurement error (relative error) and absolute value of relative error of both methods are shown in Figs. 9 and 10. The box-plot of relative measurement error of both methods is shown in Fig. 11.

**Table 1:** Area calculation and error rate comparison of 2D system and our method (RA = real area, AC = area calculation, AE = absolute value of relative error, MAPE = mean absolute percent error, var = variance)

No.	RA(*cm*^2^)	AC(*cm*^2^)		AE(%)		No.	RA(*cm*^2^)	AC(*cm*^2^)		AE(%)	
		2D	ours	2D	ours			2D	ours	2D	ours
1	2.02	2.1456	1.9641	6.22	2.77	60	0.78	0.8173	0.7533	4.78	3.43
2	0.27	0.1544	0.2581	42.82	4.41	61	3.03	3.1793	2.9266	4.93	3.41
3	1.90	1.9257	1.8883	1.35	0.61	62	4.35	4.3271	4.3493	0.53	0.02
4	5.03	2.9112	5.2029	42.12	3.44	63	3.67	5.4157	3.5271	47.57	3.89
5	9.91	9.4237	9.9797	4.91	0.70	64	1.25	1.1972	1.2581	4.23	0.65
6	0.20	0.1820	0.2078	9.00	3.89	65	3.64	3.6731	3.6411	0.91	0.03
7	1.33	1.2343	1.3485	7.19	1.39	66	1.85	2.4991	1.8362	35.08	0.75
8	12.50	7.5514	13.2206	39.59	5.76	67	0.39	0.4435	0.3890	13.71	0.26
9	1.09	1.0202	1.1145	6.41	2.25	68	0.15	0.1520	0.1450	1.35	3.32
10	0.11	0.1015	0.1065	7.74	3.19	69	0.61	0.6762	0.5812	10.84	4.73
11	0.99	0.6230	1.0458	37.07	5.64	70	1.12	1.1793	1.1410	5.29	1.98
12	6.74	6.9441	6.6396	3.03	1.49	71	0.18	0.1984	0.1795	10.22	0.28
13	0.23	0.2195	0.2347	4.56	2.04	72	0.89	0.7812	0.9094	12.22	2.18
14	0.23	0.2355	0.2255	2.40	1.95	73	0.28	0.2810	0.2825	0.34	0.91
15	6.20	5.4769	6.3677	11.66	2.70	74	0.34	0.2691	0.3529	20.84	3.79
16	0.59	0.5509	0.6075	6.63	2.97	75	0.96	0.4828	0.9796	49.70	2.04
17	0.88	0.8151	0.9091	7.38	3.30	76	2.72	1.6988	2.8421	37.54	4.49
18	3.99	4.0891	3.9113	2.48	1.97	77	1.48	0.8552	1.5444	42.22	4.35
19	0.72	0.8002	0.7487	11.14	3.99	78	3.05	4.0327	2.9880	32.22	2.03
20	2.98	3.0852	2.9614	3.53	0.62	79	1.61	0.9655	1.5669	40.03	2.68
21	1.80	2.7304	1.7449	51.69	3.06	80	0.36	0.1964	0.3722	45.45	3.39
22	2.04	1.9410	2.0404	4.85	0.02	81	2.79	2.2028	2.8371	21.05	1.69
23	2.47	2.6954	2.4928	9.13	0.92	82	2.20	1.3042	2.3150	40.72	5.23
24	5.96	5.0697	6.0596	14.94	1.67	83	1.66	1.6802	1.6637	1.22	0.22
25	3.06	2.1524	3.1713	29.66	3.64	84	1.69	1.2231	1.7594	27.63	4.11
26	0.97	0.7183	1.0164	25.94	4.78	85	4.36	4.7699	4.1851	9.40	4.01
27	3.49	3.4324	3.4955	1.65	0.16	86	2.50	2.5650	2.5028	2.60	0.11
28	12.14	12.6043	12.1365	3.82	0.03	87	2.29	2.1020	2.3011	8.21	0.49
29	4.65	3.9788	4.7489	14.44	2.13	88	5.12	5.5105	5.0762	7.63	0.85
30	0.84	0.6692	0.8794	20.34	4.69	89	0.41	0.5473	0.3989	33.49	2.70
31	0.89	0.8189	0.9227	7.99	3.67	90	8.10	9.5860	7.9691	18.35	1.62
32	8.17	6.0788	8.5622	25.60	4.80	91	0.38	0.5559	0.3731	46.29	1.82
33	1.40	1.4173	1.3779	1.24	1.58	92	2.29	2.0965	2.2347	8.45	2.41
34	1.37	1.2933	1.3213	5.60	3.56	93	0.45	0.2244	0.4401	50.14	2.20
35	0.74	0.6854	0.7051	7.38	4.71	94	0.76	0.7058	0.7765	7.13	2.17
36	1.35	1.1543	1.4025	14.50	3.89	95	8.03	7.9612	8.1409	0.86	1.38
37	0.59	0.4972	1.6038	15.73	2.34	96	3.25	3.7312	3.2752	14.71	0.78
38	5.10	3.6088	5.3373	29.24	4.65	97	11.26	11.8617	11.2349	5.23	0.22
39	1.44	1.3620	1.4128	5.42	1.89	98	3.78	3.8444	3.7817	1.70	0.04
40	0.11	0.1249	0.1131	13.57	2.85	99	0.80	1.0795	0.7856	34.93	1.80
41	0.89	0.8149	0.9306	8.44	4.57	100	0.47	0.5556	0.4623	18.21	1.65
42	2.14	2.0095	2.1400	6.10	0.00	101	1.00	1.0367	1.0077	3.67	0.77
43	0.27	0.2066	0.2830	23.49	4.83	102	0.46	0.5045	0.4387	9.66	4.62
44	4.41	4.1795	4.5546	5.23	3.28	103	0.50	0.3250	0.5174	35.00	3.49
45	2.25	1.7818	2.2985	20.81	2.15	104	6.98	5.5115	7.0106	21.04	0.44
46	1.59	1.1074	1.6206	30.35	1.92	105	2.28	1.4454	2.3099	36.60	1.31
47	1.04	1.6420	1.0235	57.88	1.58	106	0.31	0.2947	0.3106	4.92	0.20
48	2.02	2.3111	1.9642	14.41	2.76	107	0.35	0.3352	0.3586	4.23	2.46
49	2.13	2.4079	2.0971	13.05	1.55	108	4.73	4.9577	4.5500	4.81	3.81
50	2.64	2.4554	2.6605	6.99	0.78	109	0.45	0.40.5	0.4575	10.33	1.66
51	0.15	0.2385	0.1383	59.03	7.83	110	0.22	0.2280	0.2019	3.66	8.23
52	0.23	0.1250	0.2439	45.66	6.03	111	0.50	0.4164	0.5394	16.72	7.87
53	0.57	0.5687	0.5419	0.24	4.93	112	0.64	0.9773	0.5563	52.71	13.08
54	1.11	0.8933	1.2041	19.53	8.47	113	1.36	0.9864	1.4244	27.47	4.74
55	1.59	1.2239	1.6628	23.02	4.58	114	1.62	2.4508	1.5403	51.28	4.92
56	1.67	2.3435	1.5873	40.33	4.95	115	2.30	1.3004	2.4115	43.46	4.85
57	2.34	2.9862	2.2232	27.61	4.99	116	3.13	4.1409	2.9426	32.30	5.99
58	3.49	4.4483	3.3186	27.46	4.91	117	4.18	3.3670	4.3813	19.45	4.82
59	6.91	7.8040	6.5722	12.94	4.89	118	8.53	7.4215	8.9107	12.99	4.46

MAPE(2D) = 18.40%, MAPE(3D) = 2.94%, var(2D) = 0.0254, var(3D) = 0.0004

**Table 2:** The statistical index of 2D method and our method

Method	Pearson correlation	Standardized regression coefficient	Adjusted R square
Ours	0.999	0.895	0.998
2D method	0.961	0.110	0.924

From these results the measurements of the 2D method are not ideal for areas with large body curvatures. The average error rate for the 2D method is 18.40%, while the average error rate for our method is only 2.94%. In the case of less than 1*cm*^2^, the average error rate for the 2D method is 19.40%, and the average error rate for this method is 3.66%. In the case of 1*cm*^2^ and above, the average error rate for the 2D method is 17.80%, and for our method it is 2.51%.

A Mann-Whitney U test was run to determine if there were differences in relative measurement error and in absolute value of relative error between 2D method and our method. As can be seen from Figs. 9 and 10, distributions of the relative measurement error and absolute value of relative error for 2D and ours were not similar, as assessed by visual inspection. Relative measurement error for 2D and ours were statistically significantly different, U = 5668.5, z = -2.467, p = 0.014 <0.05, using an asymptotic sampling distribution for U. And absolute value of relative error for 2D and ours were statistically significantly different as well, U = 1753.5.5, z = -9.932, p = 0.000 <0.05, using an asymptotic sampling distribution for U.

As can be seen from Fig. 11, the 2D method has 4 significant outlier while ours only have one. The sample outliers of our method are also outliers of the 2D method (no.112), and the error is much larger than that of our method. Meanwhile, it can be seen that the relative measurement error of our method is much smaller and more concentrated than that of the 2D method. This shows that our method has not only better accuracy, but also better robustness.

As can be seen from Figs. 7 and 8, the mean difference value of the 2D method is -0.1, the standard deviation of the difference value is 0.714, and the 95% consistency limit is -1.5 to 1.3.Our method had a difference of 0.01, a standard deviation of 0.112, and a 95% consistency margin of -0.21 to 0.23. Only 5 groups of the two methods and true knowledge were outside the consistency limit (5/118=4.24%), and the overall proportion was relatively small. Therefore, it can be considered that the two methods and truth value have good consistency and can be used in clinical practice. However, in terms of the difference mean and the standard deviation of the difference, the 2D method in the upper arm of the difference mean is 10 times smaller, indicating that our method is closer to the truth value. Meanwhile, the standard deviation of our difference is 6 times smaller than that of the 2D method, indicating that the difference stability is also better than that of the 2D method.

It is obvious that our method is better than the 2D method for the measurement results of a large wound, minor wound, and arbitrary shape wound, and the average accuracy rate is above 97%. The variance of the 2D method is 0.0254, while the variance of our method is only 0.0004, meaning the wound area size and shape are less of a factor for our method.

In the measurement of skin wounds, the aim of quantitative measurement is to extract the wound area from the 3D model and calculate it accurately. We use the 2D to 3D to 2D method to complete the measurement. It not only overcomes the error caused by the position of the camera and the curvature of the body to the 2D measurement method, but also guarantees the accuracy of the damage area extracted from the 3D model [33]. Therefore, our method is more accurate than the 2D method.

### Comparison using different devices and methods

Table 3 compares our method with other commonly used measurement methods, advanced commercial equipment and the state-of-the-art methods in terms of accuracy, need for calibration, risk of infection, and so on with the same dataset.

**Table 3:** The Comparison of our method, other commonly used methods and business equipments

Method	Accuracy(%)	Calibration	Infection	Angle effect	Light	Tele-medicine	Facility	Computational time reference
Ours	97.06	No	Little	No	natural light	Yes	monocular camera + PC	16min
2D method	81.60	No	Little	Yes	natural light	Yes	monocular camera + PC	1min
Ruler method	52.10	No	Little	No	natural light	No	ruler	1min
MAVIS	85.26	Yes	No	Yes	darkroom	No	MAVIS	10min
Visitrak	92.17	No	Low	No	natural light	No	Visitrak + transparent film	4min
Huang [13]	86.02	No	Low	No	natural light	No	Kinect V2 + PC	8min
Yang [8]	84.31	No	Little	Yes	natural light	Yes	monocular camera + PC + color patches	2min

It can be seen from Table 3 that the accuracy of our method is higher than other methods and devices widely used at present. In addition, our method uses non-contact photography to collect wound images without a complicated pre-calibration process and has no special requirements on light. Meanwhile, the 2D software method needs the photograph angle to be as perpendicular as possible to the wound, and stereo vision may cause matching failure. The MAVIS requires the equipment to be placed at 45 degrees to take a shot. Huang’s [13] method still has a large error in parts with a large curvature of human body as well as the Yang’s [8]. In contrast, our method is not limited by shooting angle, easy to operate, can be widely applied, and avoids wound infection and pain. Moreover, our method requires only a smartphone with an ordinary PC to complete measurement. It has practical application value and possibilities, and even can be applied to remote medical treatment.

## Discussion

The wound parts acquired from the stereo vision method are fuzzy. The stereo vision method is used to calculate the 3D coordinates of spatial points in projective geometry by means of space ray intersection. This method is relatively loose in camera calibration and correction and reduces the amount of computation. Compared with it, SFM performs better in the reconstruction of the wound 3D model and requires less equipment.

Compared with 1D and 2D measurement methods, the accuracy of our method is high, especially in areas with a large curvature. Compared with the 3D method, the accuracy of this method is the same as that of the commercial equipment while our method does not need calibration. It is harmless and has little dependence on equipment. Wound area measurement can be done using a smartphone and an ordinary computer. Moreover, this method has the potential to be applied to telemedicine. Therefore, the smartphone based 3D wound area quantitative measurement in clinical and forensic applications have great prospects, and is worth further exploration and research.

As for the resolution of the camera, different cameras can bring different results. If the camera resolution is too low, the wound boundary will become very blurred, so that neither interactive segmentation nor automatic segmentation can be completed, and accurate results cannot be obtained by digital methods. Of course, if the resolution is increased, the ability of the image to express the wound itself is also enhanced, which is undoubtedly beneficial to the wound edge segmentation.

At the same time, this method has the possibility of further improvement. First, since 3D reconstruction and interactive segmentation are involved, out method takes about 16 minutes to be completed. And 3D reconstruction based on SFM requires multi-angle image information of wound area for feature point matching and point cloud location calculation. Therefore, the more images, the better the reconstruction effect will be, and the higher the measurement accuracy will be. However, this will lead to a long operation time, and shortening the operation time of 3D reconstruction will be an urgent problem for the method in this paper. Second, although the interactive segmentation method on 2D images can bring excellent segmentation results, it consumes more energy. Due to the characteristics of clinical medicine and forensic medicine, there is still no good automatic segmentation method at present. And if the segmentation result is coarse, it is bound to affect the final result. We consulted with clinical and forensic experts. In practice, because the edge of the wound is different from the border in other pictures, the definition of the wound margins relies on the experience of medical experts. In order to make the segmentation of wound as correct as possible, we used an interactive segmentation method. In the future, deep learning method can be considered to complete the automatic segmentation of the damaged area after a large number of real injury images training, so as to save human workload and improve the measurement accuracy at the same time.

## Conclusion

In this paper, we implemented a wound measurement method based on 3D transformation and smartphone images. A smartphone is used to capture wound images, which lowers costs, lessens dependence on hardware, and avoids the risk of infection. The structure from motion method (SFM) and the least square conformal mapping method (LSCM) are introduced into the measurement of the wound area. A quantitative calculation of the 3D wound area is realized, which solving the challenges that 2D methods cannot and achieving a good accuracy of 0.97.

First, based on SFM, the 3D model of a wound is reconstructed by feature extraction, sparse reconstruction, clustering and intensive reconstruction. Then, based on LSCM, the UV of the 3D model is mapped onto a 2D plane. Finally, the interactive image segmentation method and scale conversion method are used to extract and measure the wound areas.

Our method uses a contactless smartphone camera and software processing to complete the body surface wound location from 2D to 3D to 2D. Our method overcomes the defects of traditional methods, which can cause wound infection and face human subjective factors. On the other hand, it solves the problem of human curvature and the problem of shooting angles which cannot be overcome in the 2D measurement method of a computer software system based on the wound image. Moreover, it solves the shortcomings of equipment complexity and equipment dependence in commercial settings.

## Methods

**Fig. 12:**
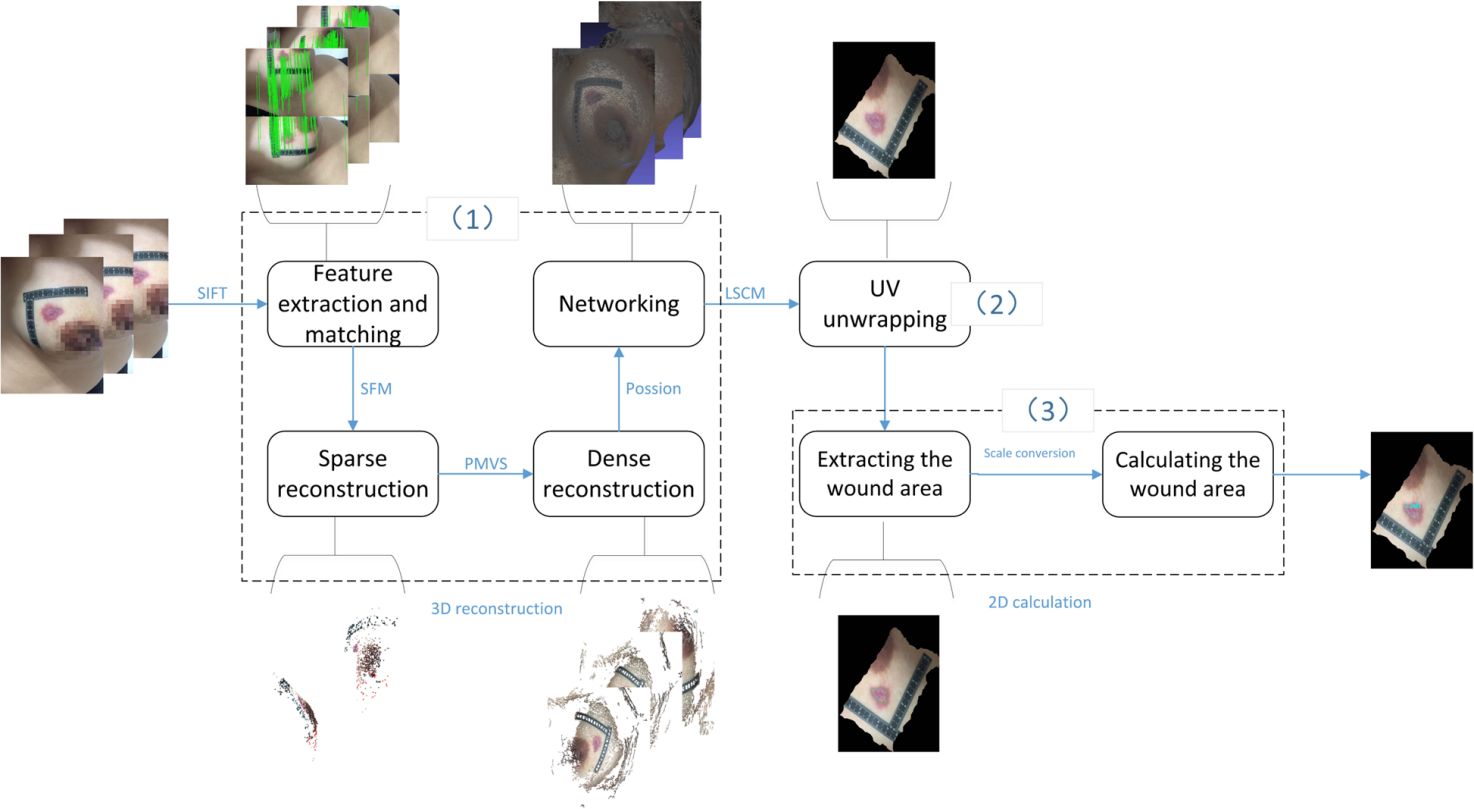
A flowchart of the proposed method. The method consists of three phases: 3D reconstruction, UV unwrapping and 2D calculation. (1) In the first phase, multiple images of one object are captured by smart-phone, the features of them are extracted and matched through SIFT. Then the 3D model of the object is reconstructed based on SFM, and goes through the process of sparse & dense reconstruction and networking. (2) In the second phase, the UV of the 3D model is unwrapped to a 2D image based on LSCM. (3) In the lase phase, the wound area on the 2D image is extracted and calculated

The main purpose of this paper is to measure the area of a surface wound precisely and quantitatively. We propose a pipeline consisting of 3D reconstruction and model mapping combined with image segmentation for measuring wound area quantitatively. The pipeline consists of three phases: (1) 3D reconstruction of the wound part of the body according to multiple images based on SFM; (2) mapping the 3D model to the 2D plane, using LSCM to do UV unwrapping (texture coordinates usually have two axes of U and V, thus called the UV coordinates); (3) we use the interactive image segmentation method and the scale conversion algorithm to extract and measure the wound area. The flowchart of the whole pipeline is shown in Fig. 12.

### 3D reconstruction based on SFM

**Fig. 13:**
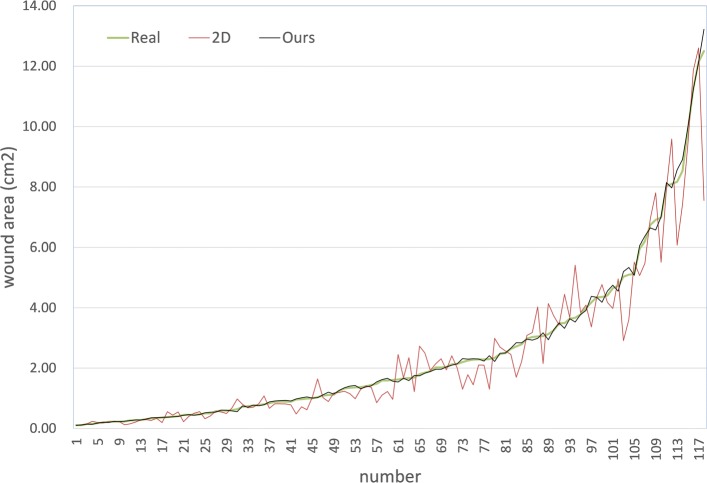
Schematic diagram of SFM. A target point *P*_1_(*x,y*,*z*) in the space passes through the horizontal, vertical, and rotational motions to point *P*_2_(*x*^∣^,*y*^∣^,*z*^∣^), point (*X,Y*) and, (*X*^∣^,*Y*^∣^) respectively represent the imaging point in a 2D plan for *P*_1_(*x,y*,*z*) and *P*_2_(*x*^∣^,*y*^∣^,*z*^∣^)

SFM [36] estimates the 3D structure from a sequence of 2D images. It first determines the spatial and geometric relationship of the target by moving the camera. It then uses the numerical method to recover 3D information by detecting the matching feature point set in multiple uncalibrated images. The schematic diagram of SFM is shown in Fig. 13. SFM extracts feature points from adjacent multiple images at different times, and establishes corresponding relationships. Then we calculate the structure and motion of the object, and generate the reconstruction of the 3D model of the sparse point cloud.

**Fig. 14:**
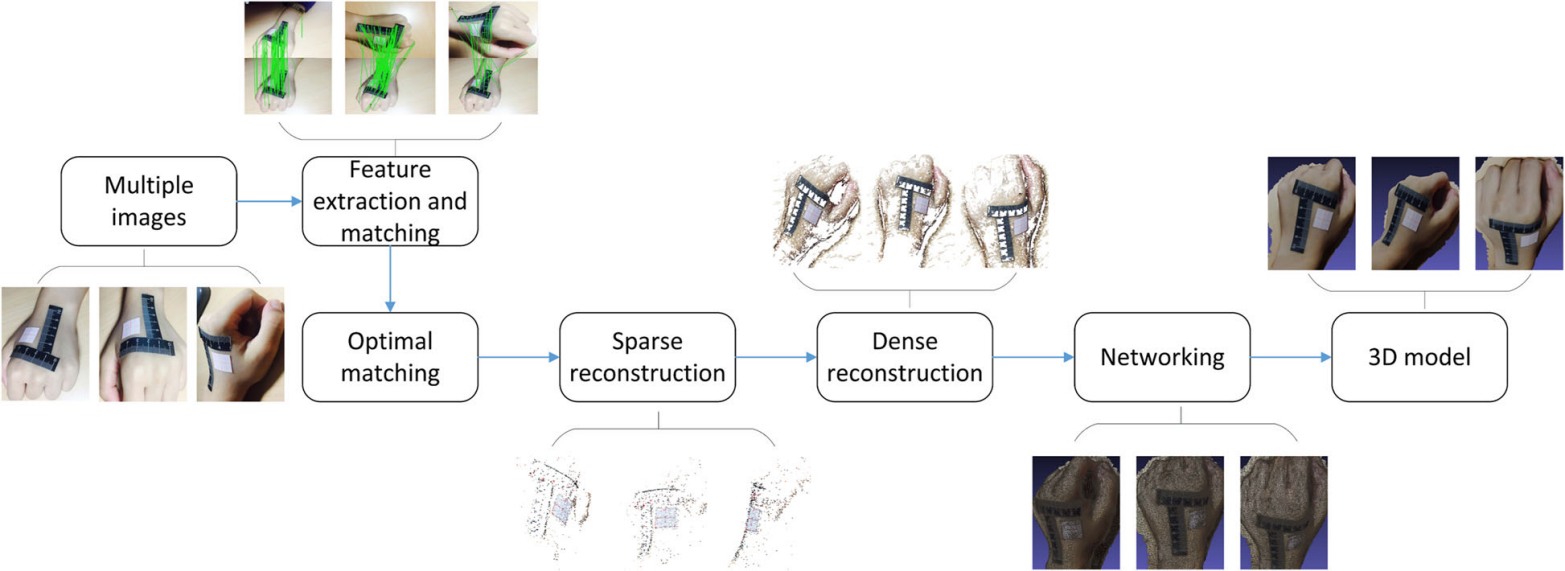
Block diagram of 3D reconstruction based on SFM. The block diagram shows the main process of 3D reconstruction. Visualization process diagrams are provided at some steps

The overall block diagram of 3D reconstruction based on structure from motion is shown in Fig. 14. We start by extracting image features using SIFT, which searches all image locations on the scale, and then uses the Gaussian differential function to identify potential interest points for scale and rotation invariance. The standard space of an image is defined as the function *L*(*x,y*,*σ*). It is usually given by the convolution of *G*(*x,y*,*σ*) with the input image *I*(*x,y*) of a sigma variable. The calculation formula is as follows:

1 $$\begin{array}{@{}rcl@{}} L(x,y, \sigma)=G(x,y, \sigma)*I(x,y),  \end{array} $$

2 $$\begin{array}{@{}rcl@{}} G(x,y, \sigma)= \frac{1}{2\pi\sigma^{2}}e^{\frac{-\left(x^{2}+y^{2}\right)}{2\sigma^{2}}},  \end{array} $$

Where, *σ* is the scale, ∗ is the convolution operation. In each candidate position, the location and scale are determined by a fitting model. We use the DOG function *D*(*x,y*,*σ*) to find out the most stable key points in the scale space. The function *D*(*x,y*,*σ*) can be evaluated on two adjacent scales. The formula is:

3 $$\begin{array}{@{}rcl@{}} D(x,y, \sigma)=(G(x,y, k\sigma)-G(x,y, \sigma))*I(x,y),  \end{array} $$

Where, *k* is a constant factor between these two scales, ∗ is the convolution operation. Based on the gradient direction of the image, each key point is assigned one or more directions. The scale of key points is used to select the Gaussian smooth image *I* with the closest scale, so that all calculations are carried out in a scale-invariant way. In this scale *σ*, for every graph sample *I*(*x,y*), the gradient size *m*(*x,y*) and direction (*x,y*) is precomputed in terms of pixel differences. We have chosen the histogram of the scale in which the key points are located and its statistical radius is 3 ×1.5 *σ*. The calculation formula of gradient size and direction is as follows:

4 $$\begin{array}{@{}rcl@{}} A=(I(x+1,y)-I(x-1,y)),  \end{array} $$

5 $$\begin{array}{@{}rcl@{}} B=(I(x,y+1)-I(x,y-1)),  \end{array} $$

6 $$\begin{array}{@{}rcl@{}} m(x,y)=\sqrt{A^{2}+B^{2}},  \end{array} $$

7 $$\begin{array}{@{}rcl@{}} \theta(x,y)={tan}^{-1}\left(\frac{B}{A}\right),  \end{array} $$

All subsequent operations on the image data are transformed by the direction, scale, and location of key points, in order to provide invariance to these transformations.

The characteristics of the images are matched according to the feature point set extracted from all relevant images. In feature matching between two images, considering image I and J are the two images, there may be a feature in image I corresponding to two characteristics in image J. In order to solve the above problems, we use F matrix and the random sampling consistency algorithm (RANSAC) [37] to optimize and filter the results after initial matching. The F matrix can associate the pixel coordinates between two images, and the pixel coordinates of each matched pair of features should be satisfied:

8 $$\begin{array}{@{}rcl@{}} \left[\begin{array}{lll} x^{\shortmid}&y^{\shortmid}&1 \end{array}\right] F \left[ \begin{array}{l} x\\ y\\ 1 \end{array} \right],  \end{array} $$

*F* is the basic matrix, (*X,Y*), and (*X*^∣^,*Y*^∣^) are the pixel coordinates of the feature points corresponding to two images, respectively.

Then, according to the matching results, the 3D reconstruction module uses SFM [38] for sparse reconstruction.

After sparse reconstruction, the collected images are clustered using clustering multi-view stereo (CMVS) [39]. CMVS can optimize the input of SFM and reduce the time and space cost of intensive matching. Then, through the patch-based multi-view stereo (PMVS) [40], each image cluster is reconstructed independently. Finally, using the Poisson surface reconstruction algorithm [41], the points are connected and networked. In this way, we set the information of the input point as a surface information model composed of a seamless triangular face, which constructs a 3D model according to the 3D point cloud.

### 3D unwrap based on LSCM

**Fig. 15:**
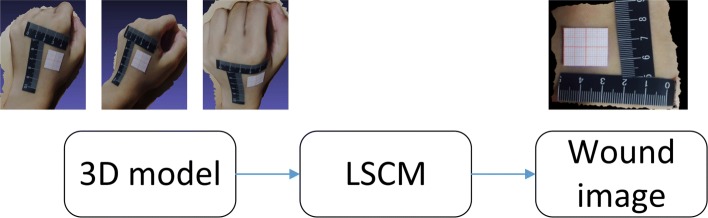
Block diagram of 3D unwrapping. The block diagram shows the main process of 3D unwrapping, and the visualization process diagrams are provided at some steps

The segmentation of a 3D model is based on two kinds of 3D models: one is the analogy of existing models [42], and the other is models from software modeling [43]. It is difficult to segment a precise local area of the model from 3D reconstruction. In order to guarantee accuracy of wound area segmentation, we adopt LSCM [33] to unwrap the surface of a 3D model onto a 2D plane. The block diagram of 3D unwrapping is shown in Fig. 15.

The conformal mapping, or conformal equivalence [44], defines a one-to-one mapping between two surfaces that preserves the local angle and local similarity. Mathematically, the conformal mapping is defined as follows: when the mapping *U* maps a domain (*u,v*) to a surface *U*(*u,v*), each (*u,v*) satisfies:

9 $$\begin{array}{@{}rcl@{}} N(u,v) \frac{\partial U(u,v)}{\partial u}= \frac{\partial U(u,v)}{\partial v},  \end{array} $$

The conformal mapping is defined on the Riemann surface. In formula (9), *N*(*u,v*) is the unit norm vector on the surface *U*(*u,v*).

**Fig. 16:**
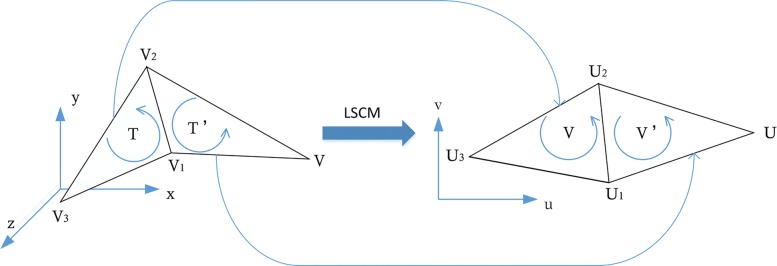
Schematic diagram of LSCM. *V* and *V*^∣^represent *T* and *T*^∣^ respectively in a 2D plane. *U*_1_,*U*_2_,*U*_3_,*U*_4_ are respectively the fixed points *V*_1_,*V*_2_,*V*_3_,*V*_4_ of the triangular section of the 3D model

LSCM [33] is a new quasi-conformal parameterization method based on a least-square approximation of the Cauchy-Riemann equations. The schematic diagram of LSCM is shown in Fig. 16. Consider a triangulation mesh *K*=(*V,T*), where *V*={*v*_1_,*v*_2_,...,*v*_*n*_},*v*_*i*_ is a set of vertex positions, and *T*={*t*_1_,*t*_2_,...,*t*_*m*_},*t*_*i*_={*v*_*i*1_,*v*_*i*2_,*v*_*i*3_} is a set of triangles consisting of triples of vertices, with *i*1,*i*2, and *i*3 denoting the vertical index in *V*. Since each triangle *t*_*i*_ has a uniquely defined norm, *t*_*i*_ can be imposed on a local orthonormal basis (*x,y*) with the normal direction along the z-axis.

Consider a triangulation mesh *K*=(*V,T*), where *V*={*v*_1_,*v*_2_,...,*v*_*n*_},*v*_*i*_ is a set of vertex positions, and *T*={*t*_1_,*t*_2_,...,*t*_*m*_},*t*_*i*_={*v*_*i*1_,*v*_*i*2_,*v*_*i*3_} is a set of triangles consisting of triples of vertices, with *i*1,*i*2, and *i*3 denoting the vertical index in *V*. Since each triangle *t*_*i*_ has a uniquely defined norm, *t*_*i*_ can be imposed on a local orthonormal basis (*x,y*) with the normal direction along the Z-axis.

Based on the Riemann equation, a mapping *U*:(*x,y*)→(*u,v*) is said to be conformal on a triangle *t*_*i*_ if and only if the following equation holds true:

10 $$\begin{array}{@{}rcl@{}} \frac{\partial U}{\partial x}+i \frac{\partial U}{\partial y}=0,  \end{array} $$

As formula (9) cannot be strictly enforced on the whole surface, the violation of the equation can be defined as the conformal energy in a square sense:

11 $$\begin{array}{@{}rcl@{}} \mathrm{E_{\text{LSCM}}} =\sum_{t_{i}\in T}\arrowvert \frac{\partial U}{\partial x}+i \frac{\partial U}{\partial y}\arrowvert^{2}A(t_{i}),  \end{array} $$

Where *A*(*t*_*i*_) is the area of the triangle *t*_*i*_.

By calculating the smallest value of $\rm {E^{}_{{LSCM}}}$ in formula (11), the planar coordinates (*u,v*) of the 3D triangle network in the parameter space is obtained, which means the 3D network is expanding in the parameters of a 2D plane.

### Wound segmentation and area calculation

**Fig. 17:**
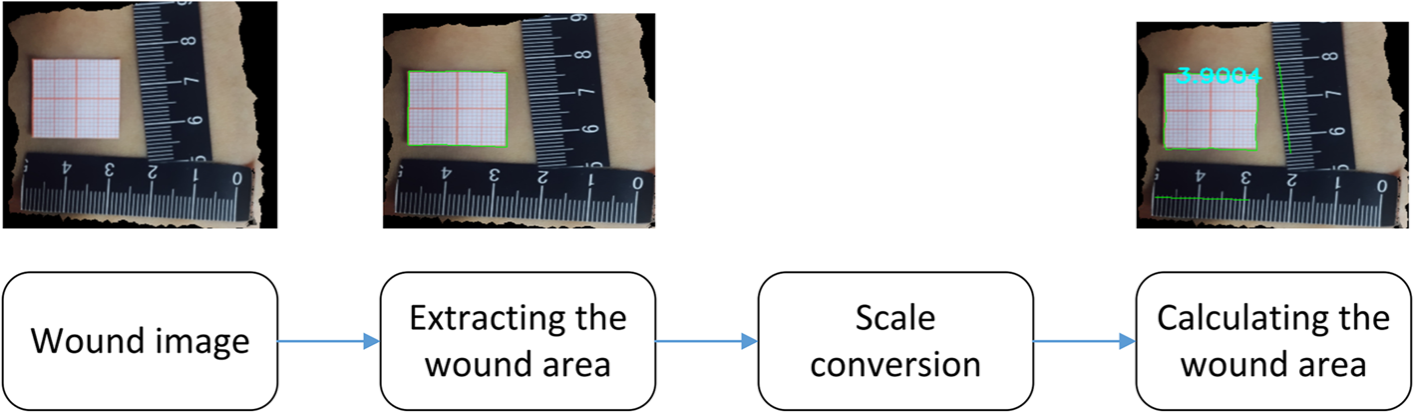
Block diagram of extraction and calculation. The block diagram shows the main process of extraction and calculation of a skin wound. Visualization process diagrams are provided at some steps

The particularity of clinical medicine requires maintaining of the authenticity of image damage. However, due to the different types of light, color and wounds, ensuring accurate segmentation of all images for the automatic segmentation method for 2D images is difficult. Therefore, we use an interactive image segmentation method to artificially modify the image segmentation results and carry out the extraction of the wound area. The wound extraction and calculation process is shown in Fig. 17.

**Fig. 18:**
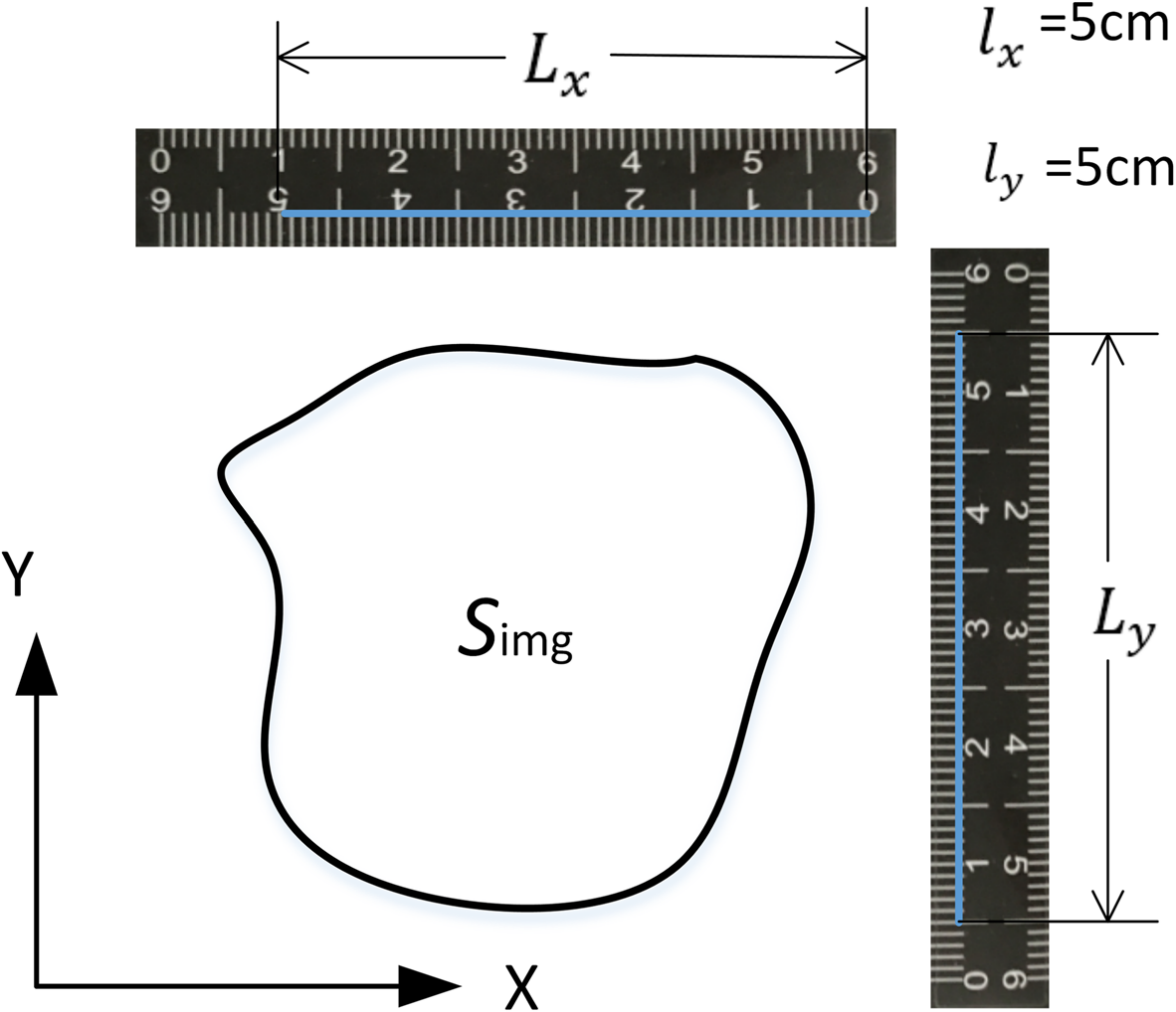
Schematic diagram of area calculation. For the example, the actual lengths $L^{}_{x}$ and $L^{}_{y}$ are 5 *cm*^2^. The pixel lengths $L^{}_{x}$ and $L^{}_{y}$ are automatically recorded by the system

We attach two lengths of known adhesive tape to the outside of the damaged area, which form X and Y directions. The user uses the mouse to mark the scale of X and Y in the image, and the system automatically labels the pixel length as $L^{}_{x}$ and $L^{}_{y}$, as shown in Fig. 18. We use the scale conversion method according to the ratio of the known length and the pixel length in X, Y direction, using formula (12) to transform the pixel area into the actual area. The measurement length is accurate to 1 *mm* and the measurement area is accurate to 1 *mm*^2^.

12 $$\begin{array}{@{}rcl@{}} \mathrm{S}_{\text{wound}}= \frac{l^{}_{x}}{L^{}_{x}}\times \frac{l^{}_{y}}{L^{}_{y}}\times \mathrm{S}_{\text{img}}. \end{array} $$

## Experiment

### Experiment setup

The experimental operating environment is a 4 core 2.00GHz CPU, 8GB memory computer. The computer visual library OpenCV and Visual Studio 2015 are used to complete the wound area measurement of our method. UV (Texture coordinates usually have U and V coordinate axes, so called UV coordinates.) unwrapping based on LSCM uses Blender open source software.

### Dataset

#### Simulated wound

**Fig. 19:**
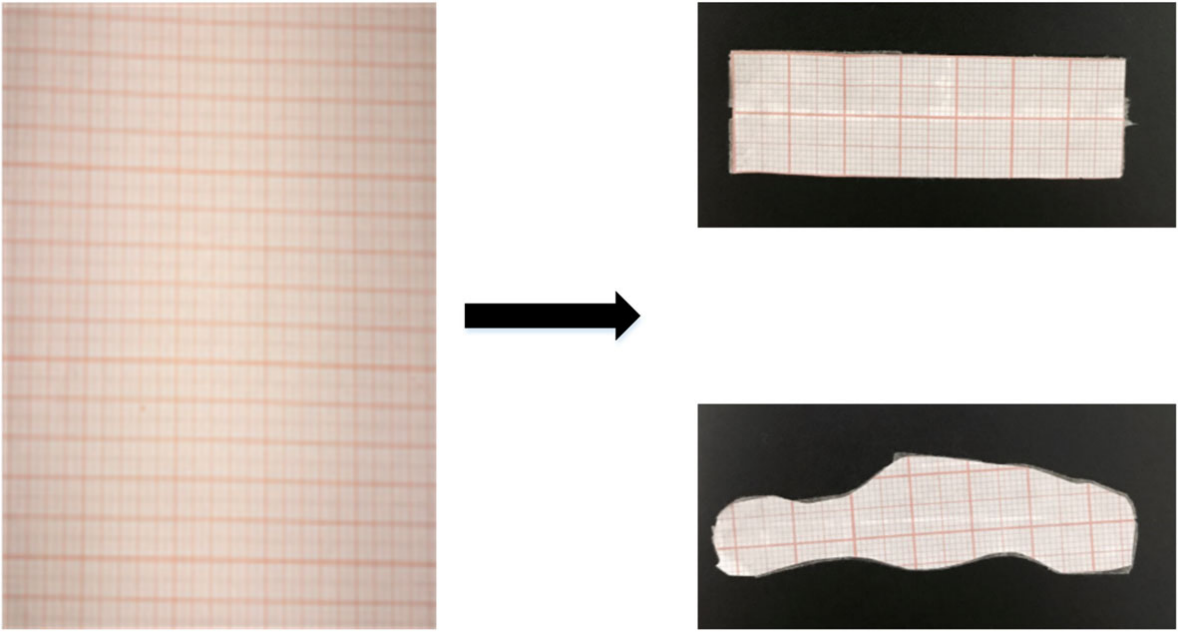
The production of simulated wound. We use scissors to cut out different shapes and sizes on the coordinate paper to simulate the wounds

Simulated wounds are used to compare the 3D reconstruction method in this paper with the popular stereo vision method. They are obtained by arbitrarily tailoring the coordinate paper. We use scissors to cut out different shapes and sizes on the coordinate paper to simulate the 2D wounds, and the cut is not in accordance with any rule. The process method is shown in Fig. 19.

The simulated wound of the rectangle and its superposition are the regular wounds, and other shape wounds are irregular wounds. Due to the more realistic significance of irregular wounds, in the experiment, there are 12 regular wounds and 28 irregular wounds. The comparison experiment attaches the simulated wounds to parts of the larger body curvatures like fingers, wrist, arm, ankle, etc.

#### Real wound

Real wounds are used to verify the accuracy of our method. They are obtained from the mammary department of the Xiyuan Hospital in China. The patients total 54 in number and range in age from 21 to 50, with a total of 118 wounds. The area of the wound ranges from 0.11 to 12.5*cm*^2^, with 44 at less than 1*cm*^2^ and 74 at 1*cm*^2^ and above. We get the wound images at multiple angles using an Iphone6 and the method above. The spatial resolution of the image is 72 dpi ×72 dpi, the color resolution of which is 3264 pixel ×2448 pixel and the bit depth is 24.

### Ground truth

The film coverage method is the most accurate measurement in the relative field. The sterile transparent film is covered in the wound area and the shape of the area is depicted artificially. Then the film is put on a coordinate paper. The area is obtained by counting the number artificially. Most researchers in the field of wound measurement use this method as the real value for wound or simulated wound area [17, 45].

The real value of the wound area in this paper is obtained by means of counting done multiple times by multiple people, and then taking the average of the counted numbers. Among them, the coordinate paper on each grid is 1*mm*^2^, and each wound is reviewed by at least 3 counters. For an incomplete grid of less than one, we artificially judge whether it is less than half of the area. When it is less than half grid, it is not calculated, otherwise, it is calculated as a whole grid.

### Implementation

#### The stereo vision method

We use an advanced 3D reconstruction device of stereo vision ZED [46] to set the baseline. ZED equipment is an advanced stereovision camera with stable results. It simulates human body parts with a simulated wound attached to the body parts with larger curvatures. We have conducted three times of parameter pre-calibration, and its mean variance is 0.0008. The pre-calibration parameters in our experiment are as follows: in the left sensor, the *fx*=1399.17,*fy*=1399.17,*cx*=983.48,*cy*=521.523,*k*1=−0.17355,*k*2=0.027811; In the right sensor, *fx*=1399.49,*fy*=1399.49,*cx*=962.345,*cy*=514.697,*k*1=−0.17177, and *k*2=0.026456; the stereo baseline =119.958, the stereo convergence =0.010710, the *rx* (*tilt*)=0.008133, the *rz* (*roll*)=0.001022. Because the ZED camera can perceive depths between 50*cm*(1.8*feet*) and 20*meters*(65*feet*), the experiences are taken from distance greater than 50*cm*. The example of 3D reconstruction results is shown in Fig. 2.

#### The 2D method

We put the adhesive tape scale next to the wound, forming an XY axis, and then shooting it with the data acquisition device in the vertical direction of the wound. Measurements are taken with close placement of the adhesive tape scale from the wound edges (0.5-1cm). When the wound is in a large part of human curvature, a picture cannot show the whole wound, we consider one wound as two wounds and shoot them vertically respectively. The images are then fed into commercial 2D measurement software, where the edges of the wound are artificially portrayed and the area of the wound is calculated. The 2D software originates from a Chinese judicial identification center, where all the people depicting the wound were doctors, legal medical experts or medical students.

#### Our method

The requirement of data acquisition equipment in our method is low. Any digital camera, smartphone, and other type of camera can be used to capture wound images. The acquisition process is not limited to the left and right movement of the acquisition equipment. It can be shot at any angle, distance, or even the same acquisition device. The device used for acquiring data is the iphone6.

We use the smartphone around the simulated wound for shooting. The angle between the two images is not greater than 30 degrees, and the number of photos is not less than 20. We keep the target fixed during shooting. Then, we use the method to reconstruct a 3D model of the simulated wound.

**Fig. 20:**
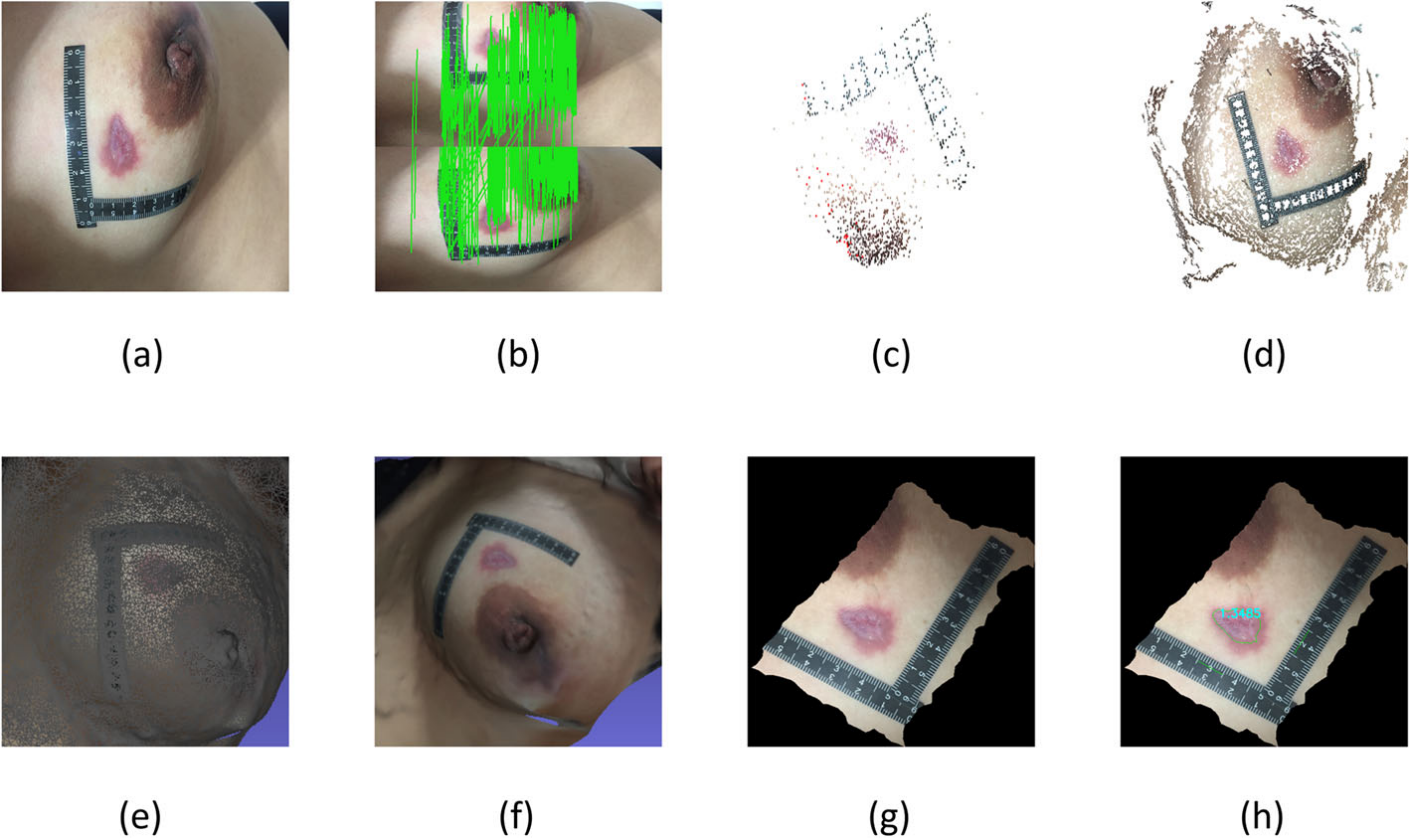
Wound area measurement process of a real wound. **a** The image captured by smart-phone. **b** The result of feature matching. **c** The spares reconstruction result. **d** The dense reconstruction result. **e** The result of networking. **f** The reconstructed 3D model. **g** The result of unwrapped images. **h** The calculated result of our method

For real wounds, we use the same method to take images and reconstruct a 3D model, and use our method to unwrap the wound area UV of the 3D model. Users trace the contour points of the whole damage area sequentially along the contour of the damaged area on the 2D image of the wound. The system selects and saves the selected points automatically, and connects each two adjacent points with a straight line. When the whole area is drawn, the system automatically connects the two points at the beginning and end, forming a closed polygon. Result for the whole process are shown in Fig. 20.

#### Ruler method

The ruler method is a simple method of wound measurement, and it is also the most used method in clinic. By measuring the length and width of the external rectangular wound with a ruler, a flexible ruler or a self-adhesive ruler, the measurement value of the wound area can be obtained by multiplying the length and width.

#### Visitrak method

The Visitrak device method is an electronic device that manually tracks the wound boundary for wound measurement. The user first describes the wound boundary with the method of film covering, and then places the film under the Visitrak transparent plate, and draws the boundary in the device interface with a pen. The device automatically calculates the length, width and area value of the wound with the Kundin formula.

## Data Availability

The datasets used and/or analysed during the current study are available from the corresponding author on reasonable request.

## Abbreviations

BA: Bundle adjustment
CMVS: Clustering multi-view stereo
GLOH: Gradient location-orientation histogram
LSCM: Least squares conformal mapping
PCA-SIFT: Principal component analysis scale-invariant feature transform
PMVS: Patch-based multi-view stereo
SBA: Sparse bundle adjustment
SFM: Structure from motion
SIFT: Scale-invariant feature transform method
SURF: Speed up robust features
